# A trend extraction method based on logistic functions and envelopes

**DOI:** 10.1038/s41598-021-04596-8

**Published:** 2022-01-11

**Authors:** Jingjing Zhang, Jinglin Luo, Xuan Zhang

**Affiliations:** 1 School of Mechatronic Engineering and Automation, Foshan University, Foshan, 528225 China; 2 Foshan Zhishi Technology Co., Ltd, Foshan, 528225 China; 3 Tianjin Aerospace Reliability Technology Co., Ltd, Tianjin, China

**Keywords:** Mechanical engineering, Applied mathematics

## Abstract

A method of step characteristic trend extraction based on logistic functions and envelopes (LFEs) is proposed in this paper. Compared with the existing trend extraction methods, the LFE method can determine the starting position of the step trend using a logistic function and extract the local trend using upper and lower envelopes. This method enhances the extraction accuracy and reduces the computation time. To verify the effectiveness of the LFE method, a simulated signal with a step trend feature was compared with the five-spot triple smoothing method, wavelet transform method and empirical mode decomposition-based method. All of these methods were applied to a real shock signal. The results demonstrate that the LFE method can reliably and accurately extract the trends with step characteristics based on less prior knowledge. Moreover, the proposed technique is suitable for industrial online applications.

## Introduction

Piezoelectric acceleration sensors usually have compact structures, high sensitivity, and high signal-to-noise ratios, and they are widely used in collecting signal processes. The application involves aviation, aerospace, navigation, and other multiple fields. However, in the practical application process, the collected signal data are accompanied by step characteristic trends in a high-overload test environment^1^, which may affect the analysis accuracy in the time domain and frequency domain. To improve this phenomenon, it is necessary to correct the signal with a trend using reasonable methods^2^.

Some methods have been studied in trend extraction, such as the five-spot triple smoothing method^3^, least-square method^4^, wavelet transform method^5^ and empirical mode decompositions (EMD)^6^. The wavelet transform method is a commonly used signal-processing method in the frequency domain. H. Vedam et al.^7^ developed a wavelet method-based nonlinear adaptive algorithm to identify trends from sensor data. Partal et al.^8^ used discrete wavelet components of measurement series to find the trend of the annual total precipitation series. However, the accuracy of the trend extraction based on wavelet transform is affected by the wavelet basis function and decomposed layer number, and these two parameter values must be determined by empirical knowledge and experimental comparison^9^. The empirical mode decomposition (EMD) method has attracted the attention of researchers because it does not require predetermining the basis function and adaptive decomposition. The essence of EMD is to decompose the signal into a set of intrinsic mode functions and the remainder^10^. In general, the signal trend can be extracted by retaining only the remainder obtained by EMD^11^. However, many signals are not monotonic, such as those with underlying trends. Hence, the remainder is not comprehensive. Yang et al.^12^ used the last several intrinsic mode functions (IMFs), where the IMFs are close to each other in the Hilbert marginal spectrum as the trend item. However, they did not give trend recognition results, such as the rise and fall. Lu et al.^9^ realized trend extraction by combining EMD with the least-square method. This method does not require empirical knowledge and complex experimental procedures to extract trend items. Dybala and Zimroz^13^ proposed a method of IMF identification based on the Pearson correlation coefficient of each IMF and the empirically determined local mean of the original signal. However, these methods do not avoid the problem of mode mixing during the EMD process. Some researchers have used the ensemble empirical mode decomposition (EEMD) to avoid the difficulty in trend extraction caused by mode mixing. However, a series of white noises will be added into the signal during the EEMD process, which can generate an extracted trend that contains nonexistent data information in the original signal. Liu and Chen^14^ proposed an EEMD-SVD method, which realized the complementary advantages of SVD and EEMD. However, the singular-value components were arranged in the order of energy reduction without considering the low complexity and high energy of the signal.

To solve these problems, this paper proposes trend extraction using the LFE method. This paper is organized as follows: in “Trend extraction by LFE method” section describes the step characteristics of the trends in signals that are collected in an impact vibration test by piezoelectric acceleration sensors. Meanwhile, this section describes how the LFE method is applied to extract trends in signals. In “Trend extraction of simulated step characteristic signals and discussion” section presents the simulation results and performance evaluation compared with other methods. In “Application in measured impact signals with step characteristics” section presents the performance evaluation of the collected test signals. In “Conclusions” section concludes the paper.

## Trend extraction by LFE method

### Signals with step characteristics trend

**Figure 1:**
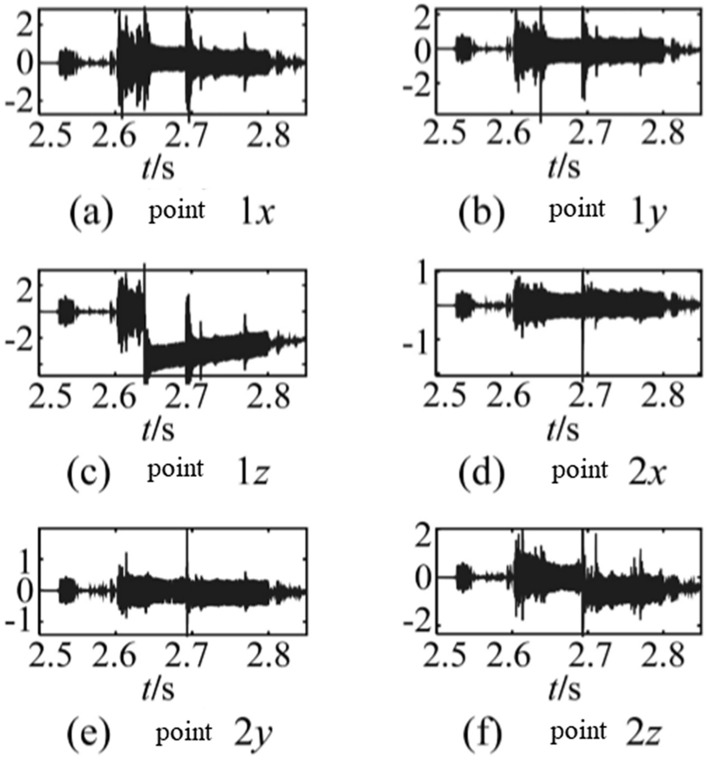
Accelerations at each measurement point from different directions. (**a**) Acceleration response signals in x direction of point 1. (**b**) Acceleration response signals in y direction of point 1. (**c**) Acceleration response signals in z direction of point 1. (**d**) Acceleration response signals in x direction of point 2. (**e**) Acceleration response signals in y direction of point 2. (**f**) Acceleration response signals in z direction of point 2.

The trends with step characteristics in signals, which are collected in an impact vibration test by piezoelectric acceleration sensors, are the result of a combination of different factors. Zhang et al.^1^ analysed the reasons for the trend appearing in high-impulsion and high-overloading conditions by acceleration sensors. Figure 1 shows the accelerations collected in a gun test at different measurement points. The accelerations of points 1 and 2 in the z direction have step characteristic trends, which are shown in Fig. 1c, f.

### Trend extraction by LFE method

Common trend extraction methods, such as the five-spot triple smoothing method, wavelet transform method and empirical mode decomposition-based method, have certain limitations in extracting the step characteristic trend in Fig. 1, which will be discussed later. This paper proposes a method using a logistic function and upper and lower envelopes to extract the step characteristic trend. The method is composed of the following steps:

Step 1: Determine the starting position of the step trend by a logistic function.

Many cases of trends occurring in high-impulsion and high-overloading conditions are shown in Fig. 1c, f. According to experience, when the baseline of the signal is drifting, the trend is usually a sudden drop, which is similar to an inverted S-shaped curve. Therefore, we select an inverted S-shaped function and calculate the mutual correlation between the S-shape function and the signal *y*(*t*). When the S-shaped function is relative to the signal with a step characteristic trend, slope *k* of the mutual correlation curves is the maximum. We can effectively determine the starting position of the step trend in this manner. The logistic function is a common S-shaped function:

1 $$P\left( t \right) = \frac{{KP_{0} e^{rt} }}{{K + P_{0} \left( {e^{rt} - 1} \right)}}$$

where $P_{0}$ is the minimum value of the curve, $K$ is the maximum value of the curve, and $r$ is the logistic growth rate or steepness of the curve.

Step 2: Identify all local extremes (maxima and minima) of *y*’(*t*_*1*_), which consists of the signals from the starting position of the step characteristics trend to the end. Meanwhile, define the rest of signals *y*(*t*) as *y*’(*t*_*0*_).

Step 3: Connect every two neighbouring local maxima (minima) by a linear equation to determine the upper envelope *E*_*max*_(*t*_*1*_) (the lower envelope *E*_*min*_(*t*_*1*_)).

Step 4: Construct the mean of the empirically determined upper and lower envelopes *m(t)* = (*E*_*max*_(*t*_*1*_) + *E*_*min*_(*t*_*1*_))/2.

Step 5: Take *m(t)* as the new signals and repeat steps 2–4 until there are fewer local extremes than the preset number. The final signals are the trend with step characteristics *r*’(*t*_*1*_).

Step 6: Define *y*_*1*_(*t*_*1*_) = *y*’(*t*_*1*_)- *r*’(*t*_*1*_) and *y*’ = [ *y*’(*t*_*0*_) *y*_*1*_(*t*_*1*_)], which are the signals without a step characteristic trend.

## Trend extraction of simulated step characteristic signals and discussion

**Figure 2:**
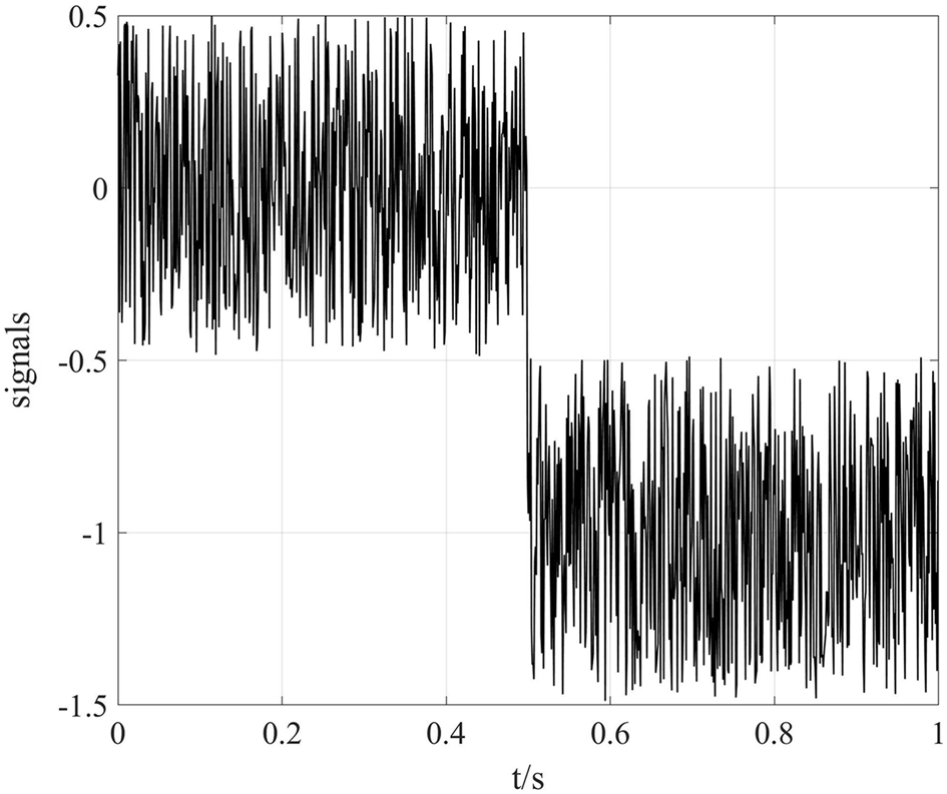
Simulated signals *y*^’^(*t*) with a step trend item.

Consider the composite signal (shown in Fig. 2),

2 $$y\left( t \right) = x\left( t \right) + n\left( t \right)$$

where *x(t)* is a Gaussian distributed random signal 1 s long, and the sampling frequency of *x(t)* is 1024 Hz; *n(t)* is the simulated trend with step characteristics.

3 $$n\left( t \right) = \left\{ {\begin{array}{*{20}c} 0 & {t < 0.5} \\ { - 1} & {t \ge 0.5} \\ \end{array} } \right.$$

**Figure 3:**
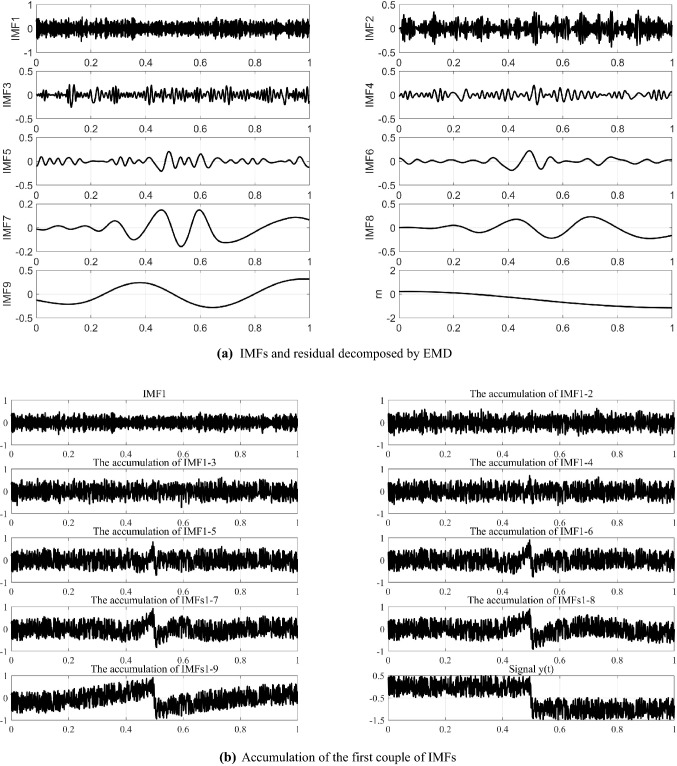
EMD method.

The simulated trend in Fig. 2 was extracted via the five-spot triple smoothing method, wavelet transform method and EMD-based method. In this simulation, the five-spot triple smoothing method was cycled 10,000 times to detrend *n(t)*. The wavelet transform method has selected basis function db8 and decomposed into 6 layers to detrend *n(t)*. Additionally, *y(t)* was decomposed into 9 IMFs, and a residual is shown in Fig. 3a. According to the accumulation of the first couple of IMFs (Fig. 3b), we regarded IMF5 to IMF9 and the residual term as the trend term *n(t)*.

**Figure 4:**
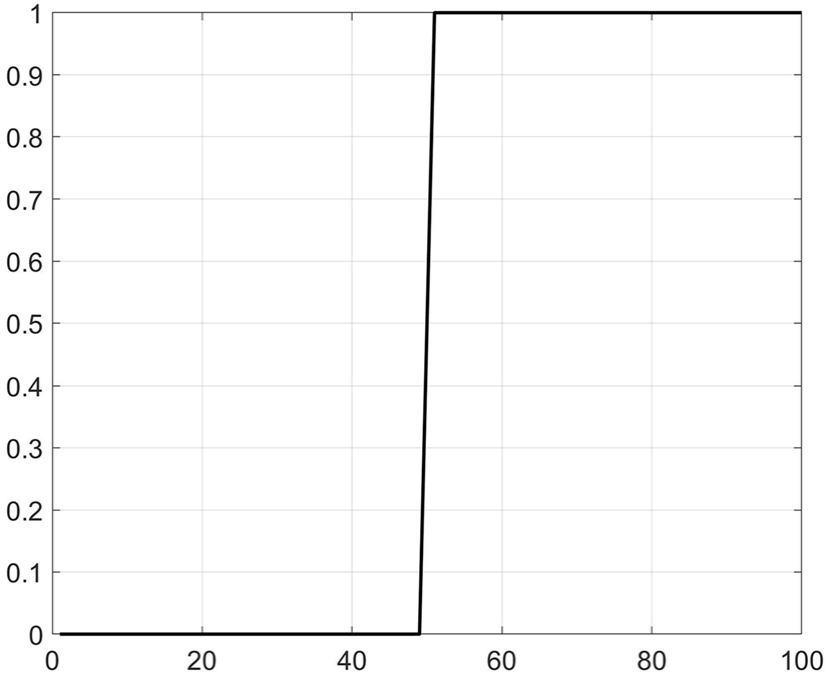
Logistic function.

**Figure 5:**
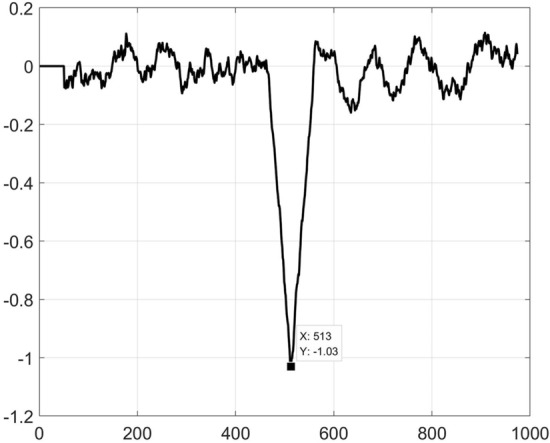
The slope of the mutual correlation curves between the logistic function and signals.

We take the method using the proposed LFE method in this paper to extract the trend *n(t)*. We take a normalized logistic function, which is shown in Fig. 4. The length of the function is 100, and the logistic growth rate *r* is 10. Therefore, we should divide signals *y(t)* composed of 1024 numbers into 924 groups. The first group *y*_*1*_ is composed of the first 100 numbers of *y(t),* and *y*_*2*_ is composed from the 2^nd^ to the 101st numbers of *y(t)*. We can similarly deduce the remaining 922 groups. Then, we calculate the slope of the mutual correlation curves between the logistic function and the 924 groups of signals *y*_*1*_-*y*_*924*_ in sequence. The 513rd group has the largest mutual correlation coefficient, which is shown in Fig. 5, and we determine the position as the starting position of the step trend. Afterwards, we identify all local extremes (maxima and minima) and make the upper and lower envelopes of the signals from the starting position of the step characteristics trend to the end. Finally, we extract the trend through steps 3–6 in “Trend extraction by LFE method” section.

**Figure 6:**
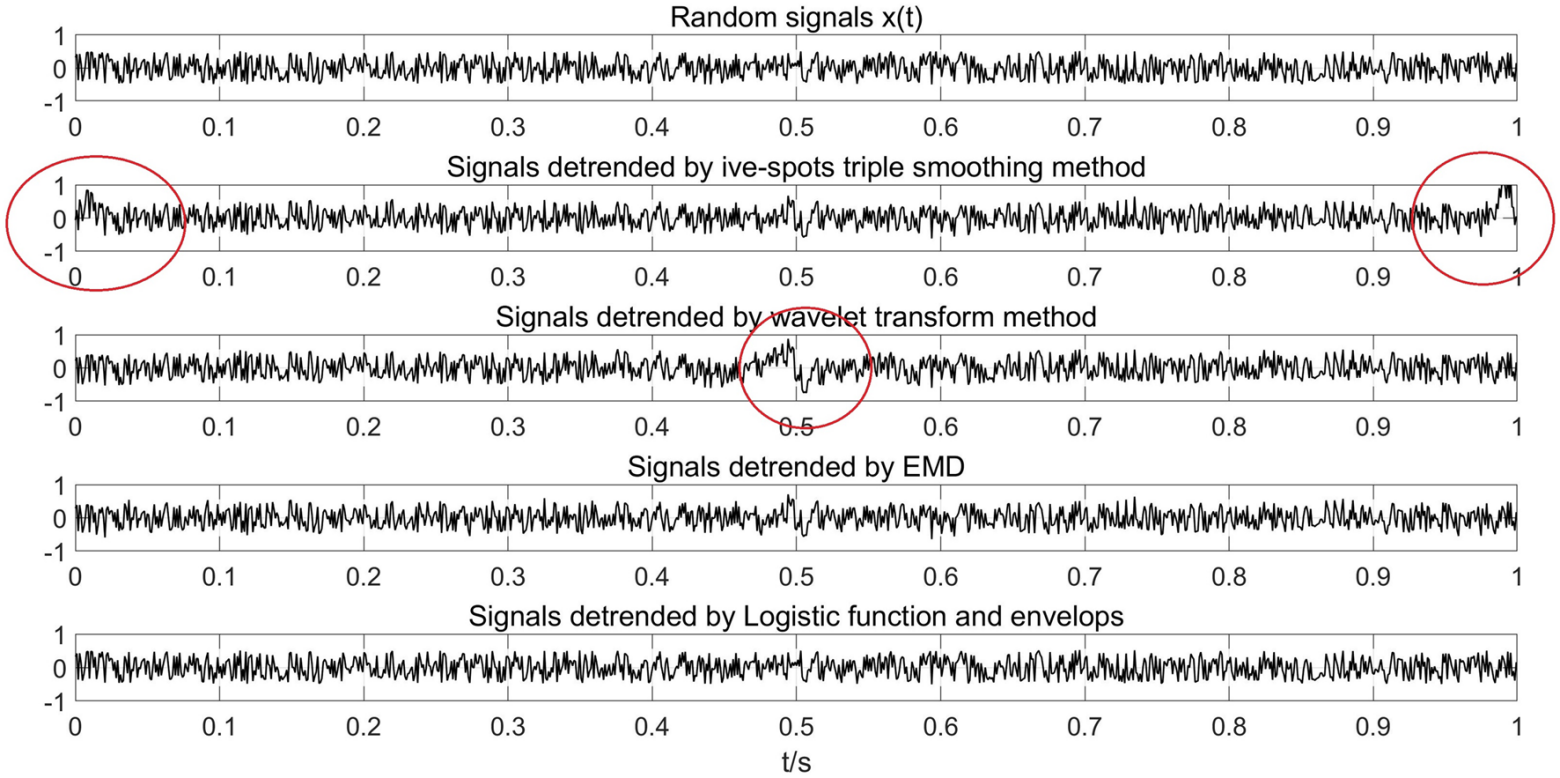
Detrended signals using different methods.

**Figure 7:**
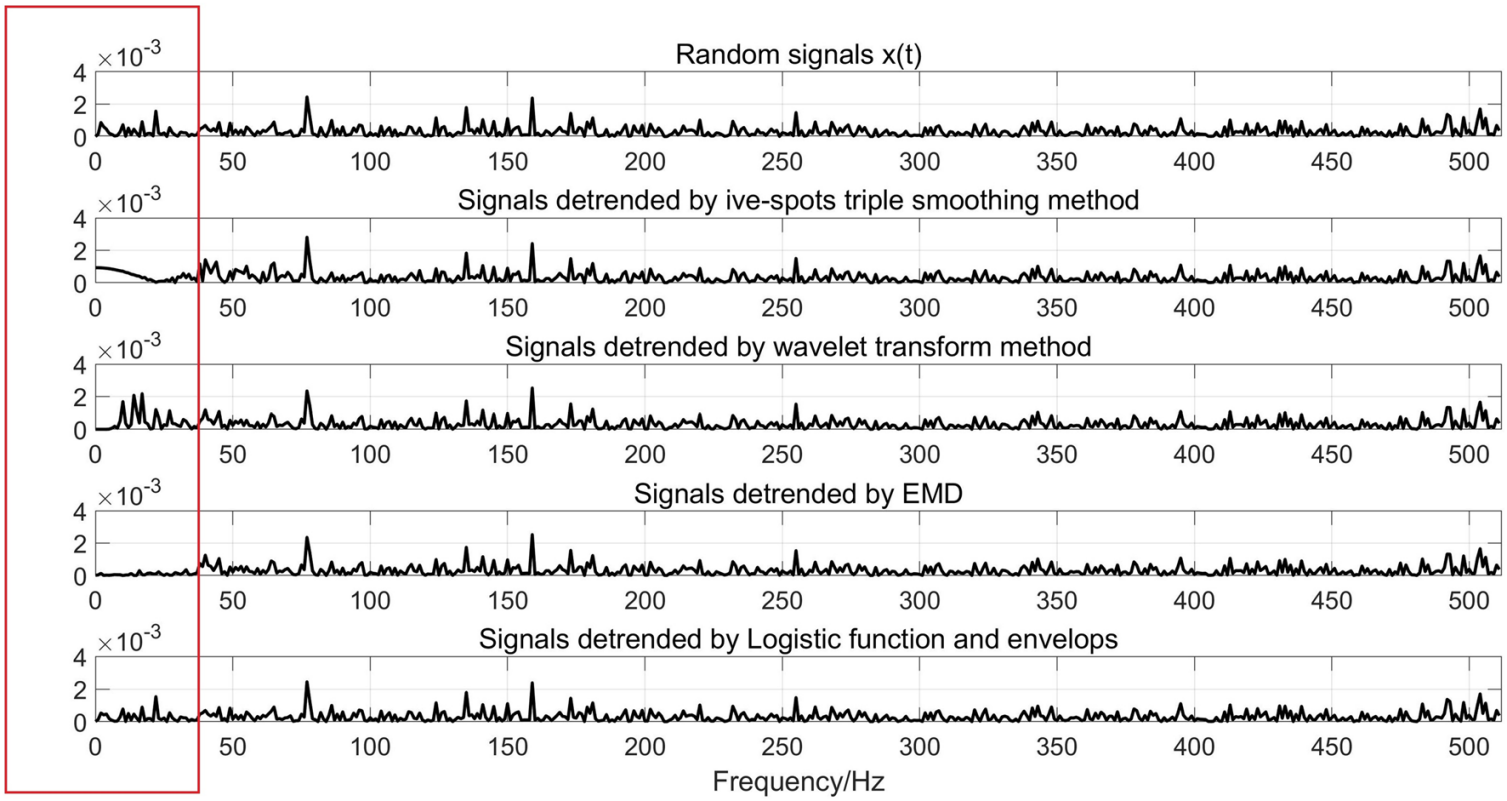
Spectrum of signals using different methods.

**Figure 8:**
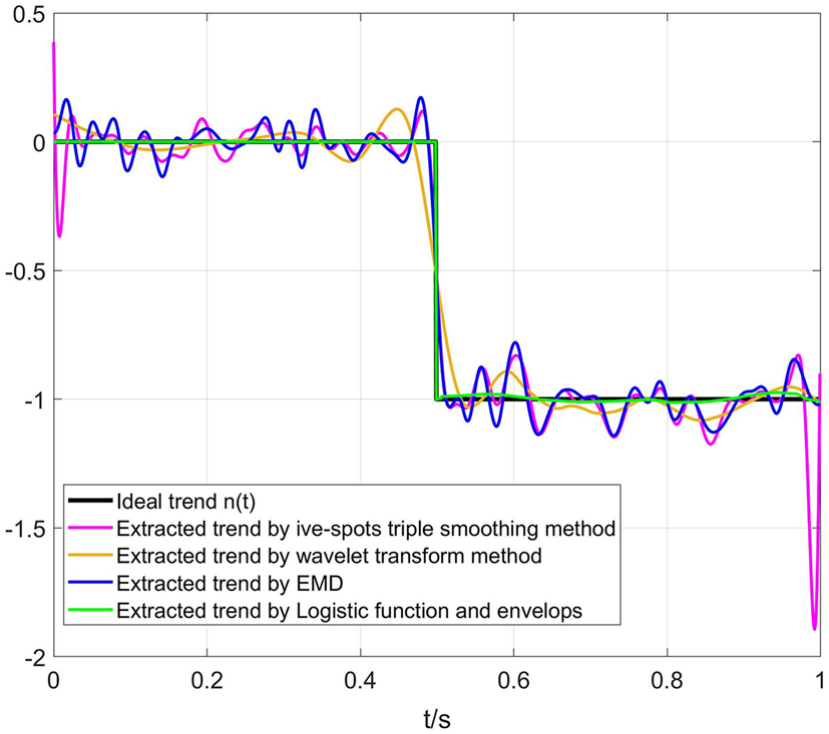
Extracted trend using different methods.

The signals *y(t)* detrended by these four methods were compared with the original random signals *x(t)*, as shown in Fig. 6. Frequency domain analysis and extracted trend item analysis were also performed. The comparison results are shown in Figs. 7 and 8. The root mean square values (RMS) of errors of the ideal trend n(t) and extracted trends with different methods are compared in Table 1.

**Table 1:** Comparisons of the RMS values of errors.

Different methods	Errors
Five-spots triple smoothing method	0.1165
Wavelet transform method	0.0802
EMD	0.0794
LFE method	0.0087

Figures 6, 7, 8 show that the trend extracted by the five-point triple smoothing method has serious end effects in the time domain. The trend extracted by the wavelet transform method has a gentle drop when t = 0.5 compared to the ideal trend, and more segments correspond to a gentle extracted trend drop when t = 0.5, which is closer to the ideal state. Therefore, the detrended signals processed by wavelet transform retain the phenomenon of trend. The trend extracted by the EMD method can effectively avoid the problems in the previous two methods. However, because of the problem of modal superposition in decomposed IMFs, some useful information of the signals has also been extracted. Figure 7 shows that the spectra of the detrended signals processed by the three approaches above at low frequency differ from the random signals *x(t)*. Fortunately, the proposed method in this paper can extract the trend with step characteristics and remain as consistent with the ideal trend as possible at the step position. The spectrum distribution law of the detrended signals is almost consistent with the random signal *x(t)*. Table 1 shows that the LFE method obtains a much smaller error than the other methods. Therefore, the proposed method in this paper can effectively extract the trend from signals with step characteristics, which are often accompanied by data acquisition in the process of overload tests.

## Application in measured impact signals with step characteristics

**Figure 9:**
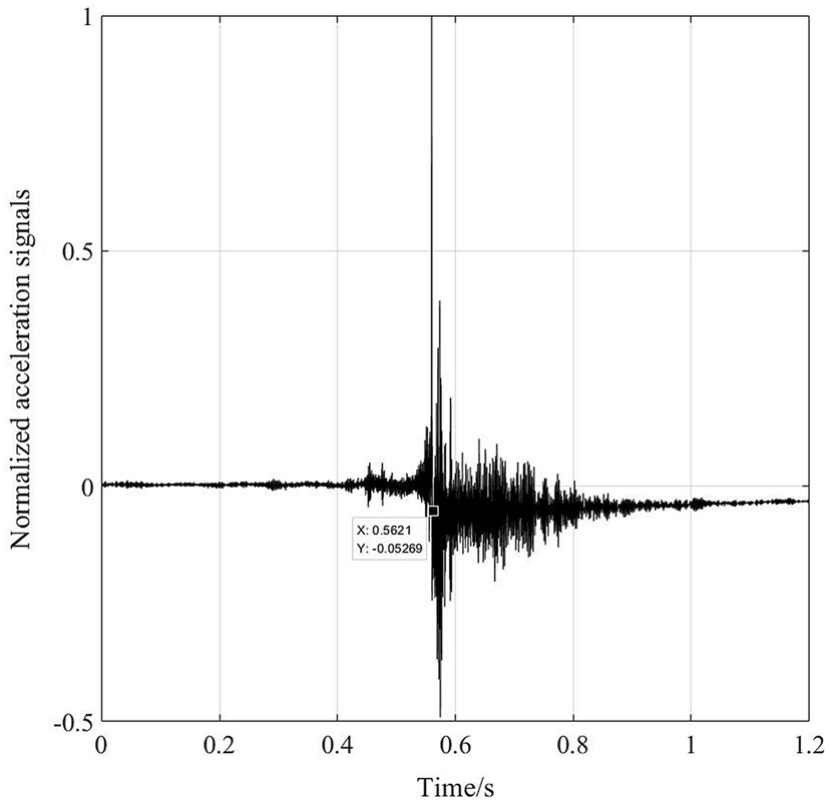
Impact signals.

**Figure 10:**
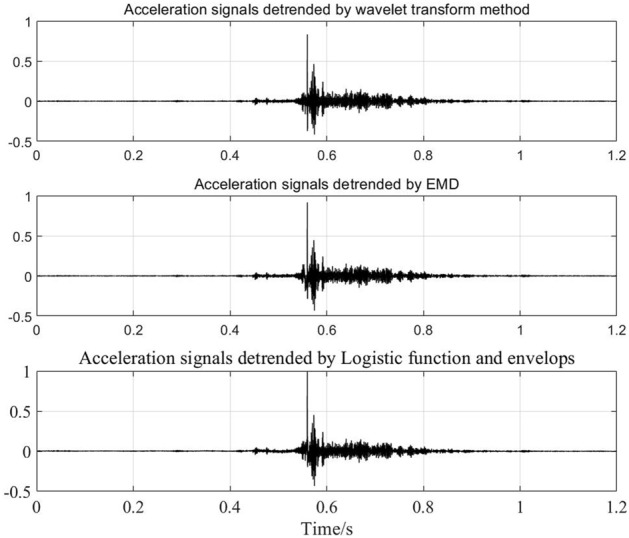
Detrended signals using different methods.

**Figure 11:**
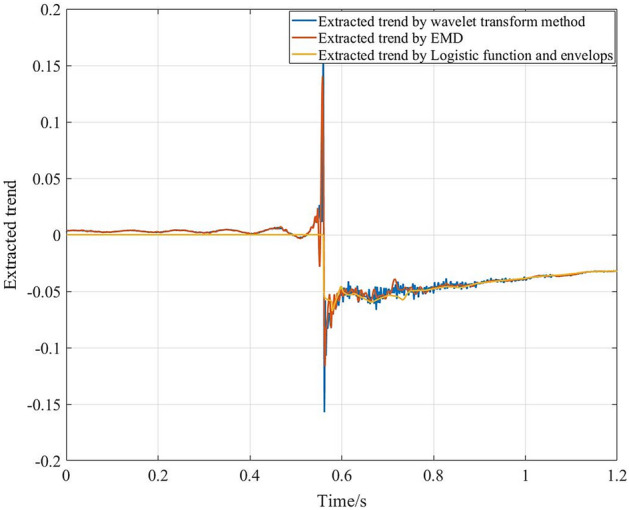
Extracted trends using different methods.

**Figure 12:**
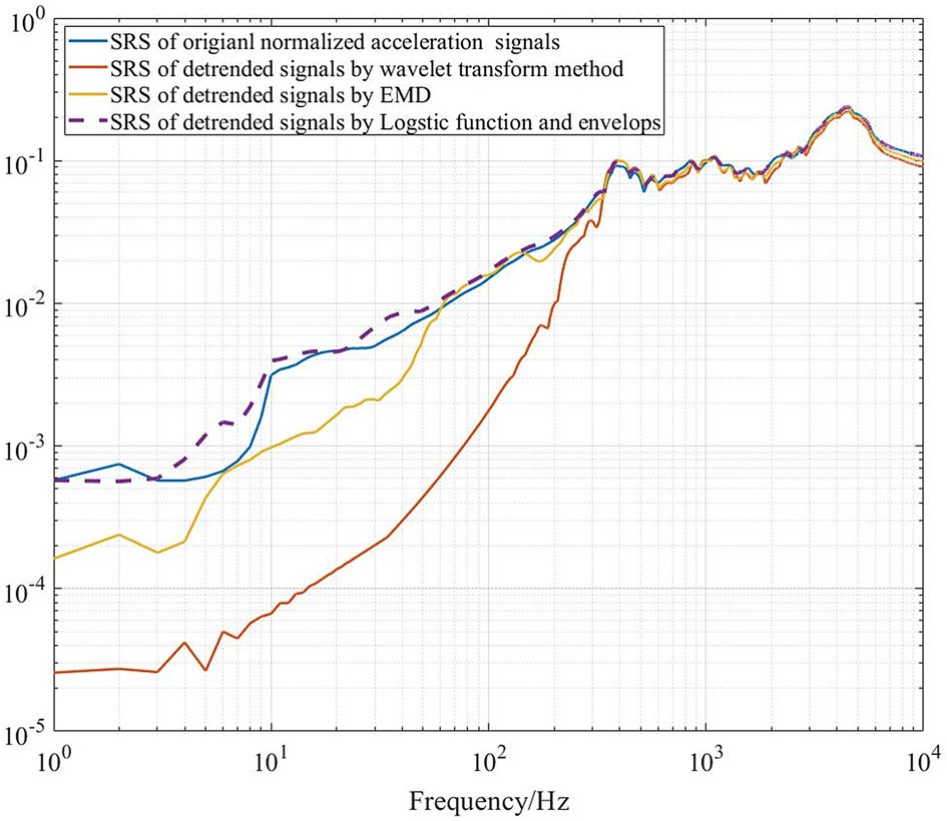
SRS of detrended signals using different methods.

Figure 9 shows normalized measured impact acceleration response signals collected by a piezoelectric acceleration sensor in a supersonic vehicle test. There is a step characteristic trend in the signals at approximately 0.56 s. The wavelet transform method, EMD and LFE method are used to extract the trend. The detrended signals obtained using different methods are shown in Fig. 10, and the extracted trends are shown in Fig. 11. The shock response spectra (SRSs) of the original signals and detrended signals using different methods are shown in Fig. 12.

Figures 10 and 11 show that all three methods can extract the trend with step characteristics. However, the extracted trends based on the wavelet transform method and EMD have some high-frequency information due to the problem of mode mixing. Hence, there are few differences between the original signals and the detrended signals due to the wavelet transform method and EMD in SRS at high frequencies. In addition, the differences in SRS at low frequencies are large. Especially in the wavelet transform method, the signal features below 350 Hz are greatly weakened. As shown in Figs. 10 and 12, compared with the other two methods, the logistic function and envelope method can effectively extract trends. Meanwhile, it can retain more signal features at both low frequencies and high frequencies.

## Conclusions

To extract the trend with step characteristics in high-overload signals, a method using the LFE method is proposed in this paper. This method uses a logistic function to identify the position of the step characteristics started and chooses the corresponding signals. The upper and lower envelopes are used to extract the trend in the corresponding signals.

Compared with the five-point triple smoothing method, the end effect of the proposed method is not obvious. Compared with the wavelet transform method and EMD, these two methods can extract trends in measured signals well. However, the differences in the shock response spectrum at low frequencies are large between the original signals and detrended signals. In addition, there are few differences between the original signals and detrended signals due to mode mixing.

The method based on the LFE method can greatly reduce the distortion of low-frequency signals and maintain consistent features with the original signals at high frequencies. Simultaneously, this method can avoid the prediction problem of wavelet transform and EMD and reduce the influence of mode mixing in them to retain as much useful information of the original signal as possible.
